# Intrinsic Resistance of *Burkholderia cepacia* Complex to Benzalkonium Chloride

**DOI:** 10.1128/mBio.01716-16

**Published:** 2016-11-22

**Authors:** Youngbeom Ahn, Jeong Myeong Kim, Ohgew Kweon, Seong-Jae Kim, Richard C. Jones, Kellie Woodling, Gonçalo Gamboa da Costa, John J. LiPuma, David Hussong, Bernard S. Marasa, Carl E. Cerniglia

**Affiliations:** a Division of Microbiology, National Center for Toxicological Research, U.S. Food and Drug Administration, Jefferson, Arkansas, USA; b MS, Bioworks LLC, Ann Arbor, Michigan, USA; c Division of Biochemical Toxicology, National Center for Toxicological Research, U.S. Food and Drug Administration, Jefferson, Arkansas, USA; d Department of Pediatrics & Communicable Diseases, University of Michigan, Ann Arbor, Michigan, USA; e Office of Pharmaceutical Science, Center for Drug Evaluation and Research, U.S. Food and Drug Administration, Silver Spring, Maryland, USA; f Division of Microbiology Assessment, Office of Pharmaceutical Quality, Center for Drug Evaluation and Research, U.S. Food and Drug Administration, Silver Spring, Maryland, USA

## Abstract

Pharmaceutical products that are contaminated with *Burkholderia cepacia* complex (BCC) bacteria may pose serious consequences to vulnerable patients. Benzyldimethylalkylammonium chloride (BZK) cationic surfactants are extensively used in medical applications and have been implicated in the coselection of antimicrobial resistance. The ability of BCC to degrade BZK, tetradecyldimethylbenzylammonium chloride (C_14_BDMA-Cl), dodecyldimethylbenzylammonium chloride (C_12_BDMA-Cl), decyldimethylbenzylammonium chloride (C_10_BDMA-Cl), hexyldimethylbenzylammonium chloride, and benzyltrimethylammonium chloride was determined by incubation in 1/10-diluted tryptic soy broth (TSB) to determine if BCC bacteria have the ability to survive and inactivate these disinfectants. With BZK, C_14_BDMA-Cl, and C_12_BDMA-Cl, inhibition of the growth of 20 BCC strains was observed in disinfectant solutions that ranged from 64 to 256 µg/ml. The efflux pump inhibitor carbonyl cyanide *m*-chlorophenylhydrazone increased the sensitivity of bacteria to 64 µg/ml BZK. The 20 BCC strains grew well in 1/10-diluted TSB medium with BZK, C_12_BDMA-Cl, and C_10_BDMA-Cl; they absorbed and degraded the compounds in 7 days. Formation of benzyldimethylamine and benzylmethylamine as the initial metabolites suggested that the cleavage of the C alkyl-N bond occurred as the first step of BZK degradation by BCC bacteria. Proteomic data confirmed the observed efflux activity and metabolic inactivation via biodegradation in terms of BZK resistance of BCC bacteria, which suggests that the two main resistance mechanisms are intrinsic and widespread.

## IMPORTANCE

Benzyldimethylalkylammonium chloride is commonly used as an antiseptic in the United States. Several recent microbial outbreaks were linked to antiseptics that were found to contain strains of the *Burkholderia cepacia* complex. *Burkholderia* species survived in antiseptics, possibly because of the degradation of antiseptic molecules or regulation of relevant gene expression. In this study, we assessed the efflux pump and the potential of *B. cepacia* complex bacteria to degrade benzyldimethylalkylammonium chloride and improved our understanding of the resistance mechanisms, by using proteomic and metabolic information. To our knowledge, this is the first systematic report of the intrinsic mechanisms of *B. cepacia* complex strain resistance to benzyldimethylalkylammonium chloride, based on the metabolic and proteomic evidence for efflux pumps and the complete biodegradation of benzyldimethylalkylammonium chloride.

## INTRODUCTION

Benzyldimethylalkylammonium chloride (BZK) is the progenitor of a group of quaternary ammonium compounds (QACs) that are commonly used worldwide in pharmaceutical formulations, cosmetics, commercial disinfectants, industrial sanitizers, and foods as preservatives, disinfectants, and stabilizers (1). Alkyldimethylbenzylammonium chloride is composed of a mixture of octyldimethylbenzylammonium chloride (C_8_BDMA-Cl) and octadecyldimethylbenzylammonium chloride (C_18_BDMA-Cl). Hexadecyldimethylbenzylammonium chloride (C_16_BDMA-Cl), tetradecyldimethylbenzylammonium chloride (C_14_BDMA-Cl), and dodecyldimethylbenzylammonium chloride (C_12_BDMA-Cl) exhibit maximum antibacterial activity (1, 2). The mechanism of bactericidal action is thought to be physical disruption and partial solubilization of bacterial cell membranes and cell walls (3, 4).

BZK solutions for hospital use are neutral to alkaline, noncorrosive on metal surfaces, nonstaining, and safe to use on all washable surfaces (2, 3). However, more than 40 outbreaks due to microbially contaminated antiseptic solutions and equipment have been reported (5–15). The probable cause of the outbreaks has been traced to the use of contaminated water in pharmaceutical processing facilities and the resulting pharmaceutical products that reduced the effectiveness of these disinfectants, linked to the storage of BZK with cotton or gauze. The common species associated with these outbreaks were *Pseudomonas* spp., *Burkholderia cepacia*, *Serratia marcescens*, and *Enterobacter* spp. (14–16). *B. cepacia* is a species within the *B. cepacia* complex (BCC), which is composed of 20 phenotypically similar but genetically distinct species. BCC species are opportunistic pathogens for humans, occasionally causing wound or urinary tract infections in immunocompromised individuals (11, 15, 17, 18). These species are especially problematic in persons with cystic fibrosis, where they can cause chronic infection of the respiratory tract (19).

The mechanisms of antibiotic resistance of BCC species have been intensively studied; however, antiseptic resistance is not completely understood. The general resistance mechanisms include adaptive phenotypic changes, efflux pumps, metabolic inactivation of biocides, and alterations of the target site (4). Efflux pumps have been best studied in *Burkholderia cenocepacia* and *Burkholderia pseudomallei* (20–24). Resistance is, in large part, attributable to efflux pump expression, mostly members of the resistance-nodulation-division (RND) family. *B. cenocepacia* strain J2315 encodes 16 RND efflux systems (25–28). Different RND efflux pumps are associated with chlorhexidine resistance in planktonic and sessile cells (29).

The major bacterium responsible for QAC degradation in activated sludge wastewater systems is *Pseudomonas fluorescens*, which initially dealkylates and thereby decreases the toxicity of QACs (30). *Aeromonas* spp. (31), *Xanthomonas* spp. (32), *Pseudomonas* spp. (30, 32–38), and environmental microbial communities (1, 37, 39, 40) also can catabolize various QACs. Bacterial transformation of BZK yields benzyldimethylamine (BDMA), benzylmethylamine (BMA), benzylamine (BA), and benzaldehyde (31, 36, 37, 41).

We hypothesized that BZK resistance could be a natural property of BCC bacteria (intrinsic resistance) or an acquired property. The objectives of this study were (i) to determine the effects of efflux pump inhibitors on the sensitivity of BCC bacteria to BZK; (ii) to assess the potential of BCC bacteria to degrade BZK and its alkyl derivatives; and (iii) to study the proteomic response to C_12_BDMA-Cl in *B. cenocepacia* AU1054 to identify resistance mechanisms. We have developed an improved understanding of the mechanisms of resistance of BCC bacteria to BZK by using proteomic and metabolic information.

## RESULTS

### Susceptibility of BCC strains to BZK alkyl derivatives.

The susceptibility of 20 strains of the BCC to BZK, C_14_BDMA-Cl, C_12_BDMA-Cl, C_10_BDMA-Cl, C_6_BDMA-Cl, and BTMA-Cl was determined (Table 1). For BZK, C_14_BDMA-Cl, and C_12_BDMA-Cl, inhibition of the growth of the BCC strains was observed at concentrations that ranged from 64 µg/ml (*B. anthina* HI2738) to 256 µg/ml (*B. cenocepacia*). BCC bacteria were still viable after 7 days of exposure to 1,024 µg/ml C_6_BDMA-Cl or BTMA-Cl. *B. cenocepacia* showed resistance to BZK, C_14_BDMA-Cl, and C_12_BDMA-Cl at 256 µg/ml, a concentration that is twice as high as that for other BCC strains. *B. anthina* HI2738 was significantly less resistant than the other BCC strains, with a minimum inhibitory concentration (MIC) range of 64 to 128 µg/ml for BZK, C_14_BDMA-Cl, C_12_BDMA-Cl, and C_10_BDMA-Cl.

**TABLE 1:** Susceptibility of BCC strains to BZK, C_14_BDMA-Cl, C_12_BDMA-Cl, C_10_BDMA-Cl, C_6_BDMA-Cl, and BTMA-Cl after 7 days

No	Strain	MICs (μg/ml) on 1/10-diluted TSB					
		BZK	C_14_BDMA-Cl	C_12_BDMA-Cl	C_10_BDMA-Cl	C_6_BDMA-Cl	BTMA-Cl
1	*B. cepacia* PC783	128	128	128	1,024	>1,024	>1,024
2	*B. cepacia* AU24442	256	128	128	1,024	>1,024	>1,024
3	*B. stabilis* AU23340	128	128	128	1,024	>1,024	>1,024
4	*B. pyrrocinnia* AU11057	128	128	128	1,024	>1,024	>1,024
5	*B. ambifaria* HI2468	128 (256)^*a*^	128	128	1,024	>1,024	>1,024
6	*B. anthina* HI2738	64 (128)	64 (128)	64 (128)	128 (512)	>1,024	>1,024
7	*B. metallica* AU0553	128	128	128	512 (1,024)	>1,024	>1,024
8	*B. metallica* AU16697	128	128	128	512 (1,024)	>1,024	>1,024
9	*B. contaminans* HI3429	128	128	128	1,024	>1,024	>1,024
10	*B. contaminans* AU24637	128	128	64 (128)	512 (1,024)	>1,024	>1,024
11	*B. diffusa* AU1075	128	128	64 (128)	512 (1,024)	>1,024	>1,024
12	*B. diffusa* AU19637	128	128	128	1,024	>1,024	>1,024
13	*B. arboris* ES0263a	256	128	128	1,024	>1,024	>1,024
14	*B. arboris* AU22095	128	128	128	1,024	>1,024	>1,024
15	*B. lata* HI4002	256	128	256	1,024	>1,024	>1,024
16	*B. cenocepacia* AU1054	128	128	128	1,024	>1,024	>1,024
17	*B. cenocepacia* AU0222	128	128	256	1,024	>1,024	>1,024
18	*B. cenocepacia* AU19236	256	256	256	1,024	>1,024	>1,024
19	*B. cenocepacia* HI2976	256	256	256	1,024	>1,024	>1,024
20	*B. cenocepacia* HI2485	128	256	256	1,024	>1,024	>1,024

a Minimum recovery concentration on 1/10-diluted TSB.

The kinetics of growth of *B. cenocepacia* HI2976 incubated at various concentrations with BZK, C_14_BDMA-Cl, C_12_BDMA-Cl, C_10_BDMA-Cl, C_6_BDMA-Cl, or BTMA-Cl were compared (see Fig. S1 in the supplemental material). Strains showed a lower rate of growth in the first 48 h of incubation in 128 µg/ml BZK, C_14_BDMA-Cl, and C_12_BDMA-Cl than in 8 to 64 µg/ml. The rate of growth increased substantially in comparison with the rate of growth in 8 to 64 µg/ml (see Fig. S1A to C). The growth rates in different concentrations of C_10_BDMA-Cl varied after 20 h (see Fig. S1D). The maximum growth population density of *B. cenocepacia* was similar in all of the C_6_BDMA-Cl and BTMA-Cl concentrations tested after 7 days of incubation (see Fig. S1E and F).

### Effect of efflux pump inhibitors on BZK.

**FIG 1:**
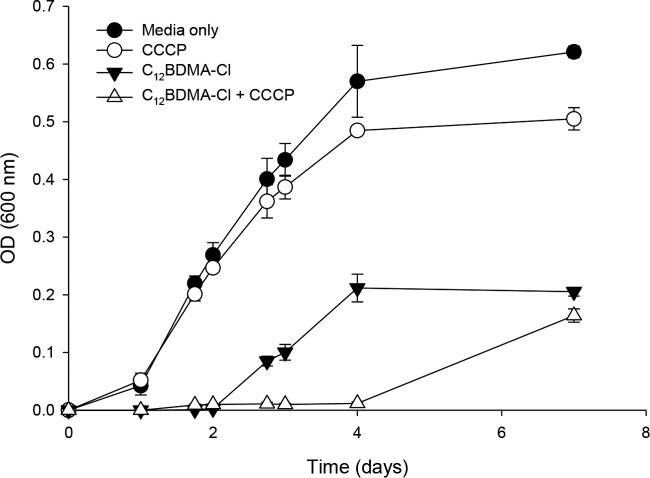
Effects of the efflux pump inhibitor CCCP on the growth of *B. cenocepacia* AU1054 in 1/10-diluted TSB medium containing 60 µg/ml C_12_BDMA-Cl.

An efflux experiment was performed to determine whether BCC bacteria were more resistant to C_12_BDMA-Cl in the presence or absence of carbonyl cyanide *m*-chlorophenylhydrazone (CCCP) because of an active efflux mechanism. *B. cenocepacia* AU1054 grew well in 1/10-diluted tryptic soy broth (TSB) supplemented with CCCP only and reached a level almost equal to that in the medium without CCCP. The strain showed a lower rate of growth in the first 4 days of incubation in the medium supplemented with CCCP and 60 µg/ml C_12_BDMA-Cl than in the medium without CCCP. This demonstrated that CCCP increased the sensitivity of *B. cenocepacia* AU1054 to C_12_BDMA-Cl in 1/10-diluted TSB (Fig. 1).

### Biodegradation of BZK and alkyl derivatives.

All 20 BCC strains grew well in 1/10-diluted TSB medium with BZK, C_12_BDMA-Cl, and C_10_BDMA-Cl and partially degraded the compounds in 7 days. Table 2 shows a comparison of BZK, C_12_BDMA-Cl, and C_10_BDMA-Cl in 1/10-diluted TSB culture sets after 7 days of incubation. The degradation of BZK ranged from 4.7 to 42.6%. The degradation of C_12_BDMA-Cl and C_10_BDMA-Cl was between 3.2 to 66.1% and 2.9 to 42.6%, respectively. *B. cenocepacia* AU1054 degraded 64 µg/ml BZK (4.7%) and 128 µg/ml C_10_BDMA-Cl (4.7%) at lower levels than the other BCC strains.

**TABLE 2:** Degradation of BZK, C_12_BDMA-Cl and C_10_BDMA-Cl by BCC strains

No.	Strain	Concentration in µg/ml (mean % ± SD)			
		BZK	C_12_BDMA-Cl	C_10_BDMA-Cl	
1	*B. cepacia* PC783	64^*a*^ (18.4 ± 0.3)^*b*^	64 (18.0 ± 1.8)	128 (18.4 ± 0.3)
2	*B. cepacia* AU24442	64 (24.0 ± 4.7)	64 (8.6 ± 0.9)	128 (24.0 ± 4.7)
3	*B. stabilis* AU23340	64 (21.7 ± 1.2)	64 (8.2 ± 2.6)	128 (21.7 ± 1.2)
4	*B. pyrrocinia* AU11057	64 (25.3 ± 2.4)	64 (6.6 ± 3.6)	128 (25.3 ± 2.4)
5	*B. ambifaria* HI2468	64 (22.4 ± 1.5)	64 (9.9 ± 1.1)	128 (22.4 ± 1.5)
6	*B. anthina* HI2738	32 (12.6 ± 1.9)	32 (6.4 ± 2.9)	128 (12.6 ± 1.9)
7	*B. metallica* AU0553	64 (22.9 ± 7.7)	64 (8.6 ± 2.2)	128 (22.9 ± 7.7)
8	*B. metallica* AU16697	64 (19.6 ± 4.4)	64 (6.1 ± 0.8)	128 (19.6 ± 4.4)
9	*B. contaminans* HI3429	64 (42.6 ± 12.3)	64 (66.1 ± 0.6)	128 (42.6 ± 1.3)
10	*B. contaminans* AU24637	64 (21.9 ± 0.4)	32 (8.5 ± 2.4)	128 (2.9 ± 0.4)
11	*B. diffusa* AU1075	64 (7.5 ± 5.8)	32 (3.2 ± 0.3)	128 (7.5 ± 5.8)
12	*B. diffusa* AU19637	64 (14.6 ± 3.8)	64 (4.3 ± 0.3)	128 (14.6 ± 3.8)
13	*B. arboris* ES0263a	64 (12.3 ± 2.8)	64 (8.1 ± 4.2)	128 (14.9 ± 0.9)
14	*B. arboris* AU22095	64 (9.6 ± 4.3)	64 (7.9 ± 2.9)	128 (9.6 ± 4.3)
15	*B. lata* HI4002	64 (11.1 ± 3.1)	64 (6.4 ± 2.5)	128 (11.1 ± 3.1)
16	*B. cenocepacia* AU1054	64 (4.7 ± 2.4)	64 (5.6 ± 0.6)	128 (4.7 ± 2.4)
17	*B. cenocepacia* AU0222	64 (6.7 ± 3.4)	64 (3.7 ± 1.5)	128 (6.7 ± 3.4)
18	*B. cenocepacia* AU19236	64 (13.5 ± 6.7)	64 (5.9 ± 2.3)	128 (13.5 ± 6.7)
19	*B. cenocepacia* HI2976	64 (10.4 ± 10.7)	64 (8.3 ± 2.3)	128 (10.4 ± 1.7)
20	*B. cenocepacia* HI2485	64 (21.9 ± 1.7)	64 (4.5 ± 0.7)	128 (21.9 ± 1.7)

a Applied concentration of BZK and alkyl derivatives for degradation.

b Percent degradation of BZK and alkyl derivatives after 7 days of incubation.

**FIG 2:**
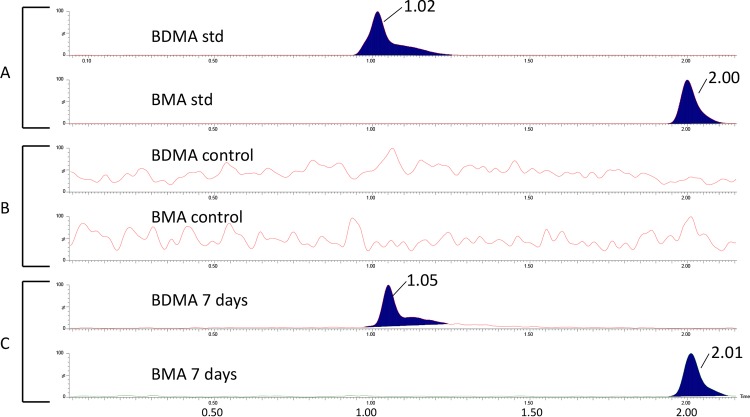
LC-MS analysis evidencing the degradation of C_12_BDMA-Cl to BDMA and BMA by *B. cenocepacia* AU1054. (A) BDMA and BMA at a standard (std) concentration of 100 ng/ml. BDMA elutes at ~1.0 min, whereas BMA elutes at ~2.0 min. Only the primary transitions are shown. However, the secondary transitions were acquired and seen for each compound. (B) Control sample without the addition of C_12_BDMA-Cl after 7 days of incubation. (C) Terminal-point sample taken after 7 days of incubation with the addition of C_12_BDMA-Cl.

Liquid chromatography (LC)-mass spectrometry (MS) analysis of methylene chloride extracts from *B. cenocepacia* AU1054 cultures incubated with 64 µg/ml C_12_BDMA-Cl was performed. Multiple reaction monitoring (MRM) ion chromatograms revealed two peaks whose primary and secondary transitions and retention times were consistent with those of authentic standards of BDMA and BMA (Fig. 2). BDMA and BMA were not detected in the control sample. Whereas only the results for 7 days of incubation are shown, similar results were found after 3 days of incubation.

### Proteome analysis of BCC bacteria in response to BZK.

**FIG 3:**
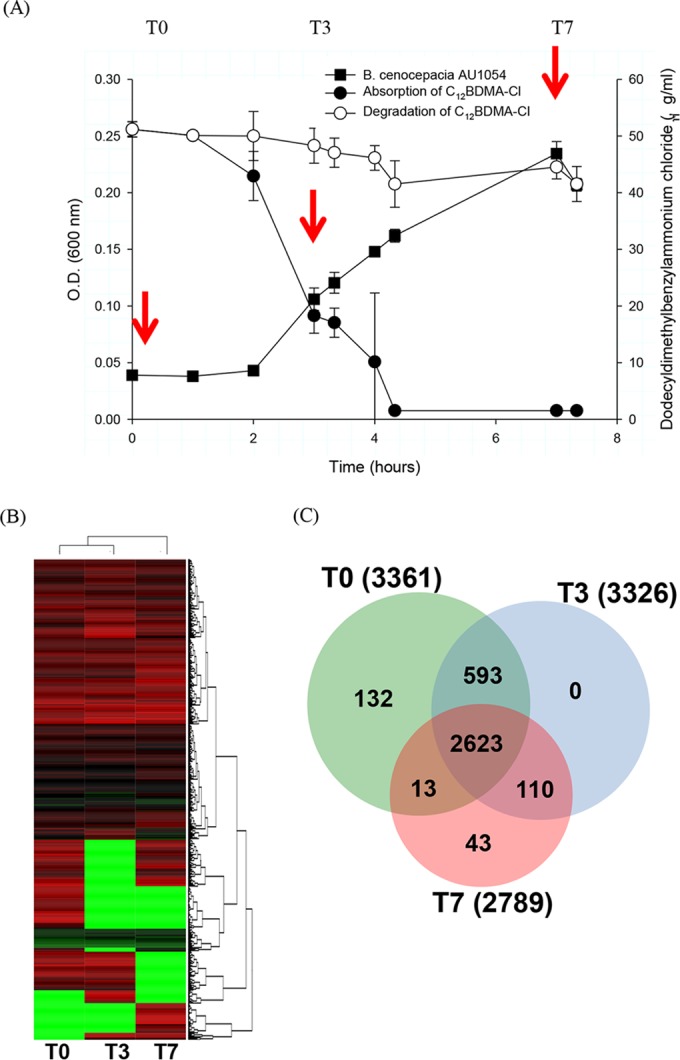
Summary of proteins identified in this proteome study. (A) Time course of sampling. (B) Cluster analysis of proteomic data sets showing the correlation between protein expression profiles and treatment time. (C) Venn diagram analysis of the proteome (3,747/6,632).

To understand the global cellular response of BCC bacteria to BZK, a high-throughput whole-cell proteome analysis was conducted. Time points were chosen on the basis of the results of the BZK metabolism experiment. As a whole, we identified 3,361, 3,326, and 2,789 proteins, from culture samples at time zero, day 3, and day 7 (Fig. 3A), respectively, which add up to 3,747 unique proteins (56.5% of the 6,632 genome-predicted proteins) in total (see Table S1 in the supplemental material). As revealed in the clustering analysis (Fig. 3B), the proteomic data set shows an apparent correlation between the profiles of protein expression with respect to BZK exposure time, indicating the reliability of the proteomic results. About 2,623 proteins were identified as commonly shared between controls and two other samples, while other proteins (1,124) were identified either in control (time zero) or BZK-exposed samples (Fig. 3B and C). Compared with the sample at time zero, about 1,648 proteins at day 3 or 7 were up- or downregulated.

**FIG 4:**
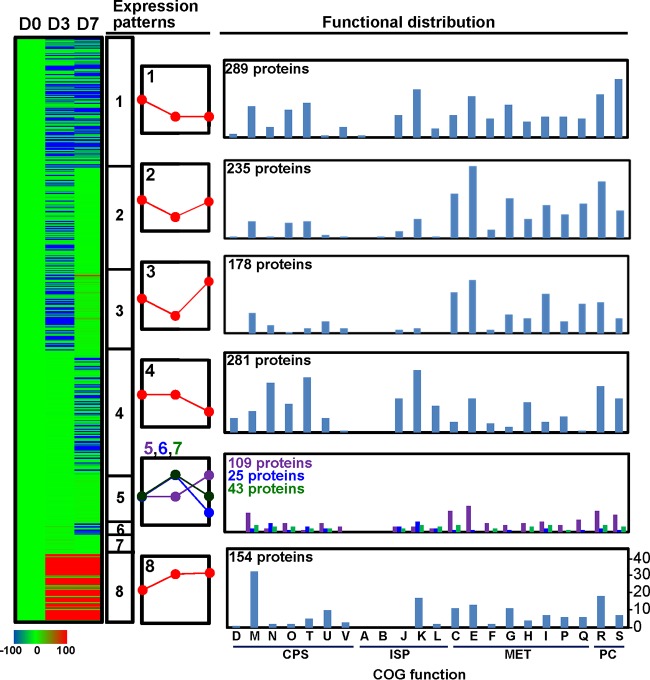
Protein expression pattern analysis by STEM (42) and functional distribution of the up- or downregulated proteins. ISP, information storage and processing; CPS, cellular processes and signaling; Met, metabolism; PC, poorly characterized. COG functional categories: D, cell cycle control, cell division, chromosome partitioning; M, cell wall/membrane/envelope biogenesis; N, cell motility; O, posttranslational modification, protein turnover, and chaperones; T, signal transduction mechanisms; U, intracellular trafficking, secretion, and vesicular transport; V, defense mechanisms; J, translation, ribosomal structure, and biogenesis; K, transcription; L, replication, recombination, and repair; C, energy production and conversion; E, amino acid transport and metabolism; F, nucleotide transport and metabolism; G, carbohydrate transport and metabolism; H, coenzyme transport and metabolism; I, lipid transport and metabolism; P, inorganic ion transport and metabolism; Q, secondary metabolite biosynthesis, transport, and catabolism; R, general function prediction only; S, function unknown.

In the short time series expression miner (STEM) analysis (42), about 1,312 proteins were initially filtered and sorted into eight significant temporal expression profiles, in which proteins were compared not only with respect to time zero but also between time points (Fig. 4; see Table S2). In general, treatment with BZK had an immediate and dynamic effect on the expression of proteins at a genome-wide level, whose functions are distributed all over the COG (Clusters of Orthologous Groups) categories in BCC bacteria. As shown in Fig. 4, about 702 proteins responsible mainly for metabolism (MET; C, E, G, I, and Q), cellular processes and signaling (CPS; N, O, and T), and information storage and processing (ISP; J and K) belong to initially downregulated STEM patterns 1 to 3, while about 222 proteins functioning mainly for MET (C, E, G, and I), CPS (M and U), and ISP (K) belong to STEM patterns 5 to 8, which were initially upregulated (Fig. 4). Proteins belonging to COG functional categories N, O, and T of CPS and J, K, and L of ISP were not initially changed in expression but later were downregulated (STEM pattern 4), while proteins involved in categories M of CPS and C, E, and Q of MET belong to STEM pattern 5 with no initial expression change but later were upregulated (Fig. 4).

### Transporters.

**FIG 5:**
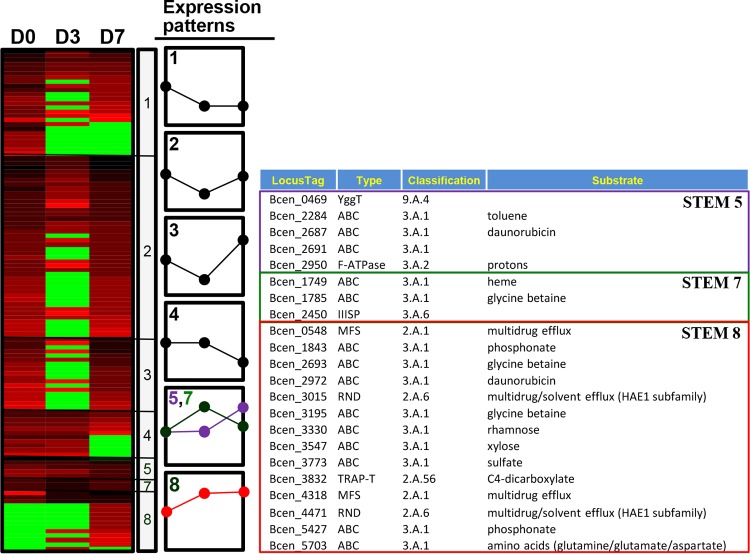
Transporter expression pattern analysis by STEM and transporter information belonging to STEM patterns 5, 7, and 8. TransportDB (65) was used for annotation and classification of transport genes from *B. cenocepacia* AU1054. D0, time zero; D3, day 3; D7, day 7.

**FIG 6:**
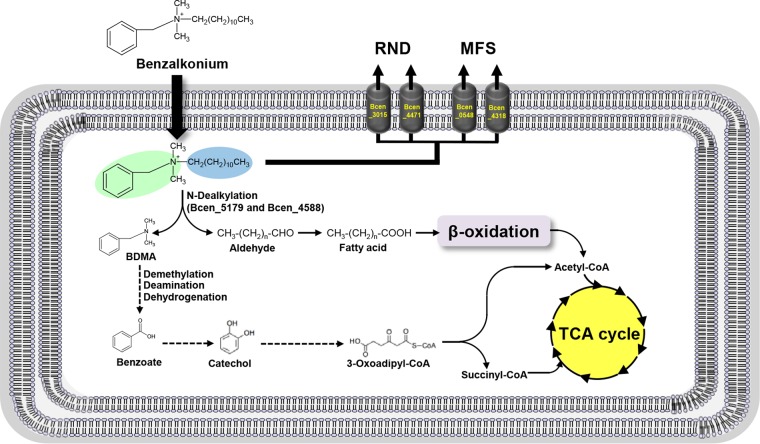
The intrinsic resistance mechanisms of efflux pumps and biodegradation of BZK in BCC strains. The aromatic and alkane groups of BZK are highlighted with green and blue ellipses, respectively. In the BZK degradation pathway, solid arrows indicate one-step reactions, while dashed arrows indicate multiple steps of enzyme reactions. TCA, tricarboxylic acid.

In the initial analysis of the *B. cenocepacia* strain AU1054 genome, about 893 genes were annotated as transporter related (see Table S3). Of these genes, about 341 have been expressed as proteins (see Table S3). As shown in Fig. 5 (see Table S3), about 117 transporter-related proteins were STEM filtered and sorted into seven STEM expression patterns, indicating dynamic responses of various transport systems to BZK. No transport proteins were identified for STEM pattern 6 with initial upregulation (day 3) but later downregulation (days 7). In general, the class I primary active transporters with substrate specificity for sugars and amino acids (or dipeptides), including the ATP-binding cassette (ABC) transporter superfamily and the P-type ATPase superfamily, were initially downregulated (STEM patterns 1 to 3, see Fig. 6; see Table S3). On the other hand, two proteins, Bcen_0548 and Bcen_4318, of the major facilitator superfamily (MFS) and two proteins, Bcen_3015 and Bcen_4471, of the RND family, functioning for multidrug efflux pumping, were continually upregulated (STEM pattern 8) (Fig. 5). In addition, more than 10 ABC-type transporters with diverse substrate specificities, including toluene, xylose, and glycine, also belong to STEM patterns 5, 7, and 8 (Fig. 5; see Table S3). In the case of porin, of the 13 STEM-filtered porin proteins, about 8 were upregulated at day 7 (STEM pattern 5) or continually upregulated (STEM pattern 8).

### Degradation enzymes potentially responsible for BZK resistance.

Genome screening was followed by proteome-based filtering to identify genes and enzymes potentially involved in the degradation of BZK by BCC bacteria. Initially, the metabolic data and bioinformatic analysis guided genome-based identification of the genes involved in BZK degradation, which includes the C-N cleavage and subsequent degradation of the corresponding BDMA and alkyl aldehyde. The whole-cell proteome of *B. cenocepacia* AU1054 incubated with BZK filtered out the static genes that are not expressed.

On the basis of the metabolic evidence (Fig. 2), the biodegradation of BZK proceeded through the cleavage of the C-N bond between BDMA and alkyl groups (N dealkylation). Two potential C-N bond cleavage enzymes responsible for the initial step of the BZK degradation pathway were identified. An amine oxidase (Bcen_5179) was constitutively expressed, and a Rieske-type oxygenase (Bcen_4588) was expressed with STEM pattern 3 (initial downregulation and later upregulation). For further degradation of BDMA, a total of eight catabolic enzymes were identified. All of the enzymes responsible for complete benzoate degradation were constitutively expressed, indicating the generation of acetyl coenzyme A (acetyl-CoA) and succinyl-CoA. Among the expressed enzymes, a catechol 1,2-dioxygenase (Bcen_1307), which catalyzes the oxidative ring cleavage of the toxic intermediate catechol to form *cis*,*cis*-muconic acid, was upregulated (STEM pattern 8). The aldehyde generated by N dealkylation of BZK also seems to be further oxidized to a fatty acid, which is conjugated to CoA and further processed by β oxidation to generate acetyl-CoA (see Fig. S2). The enzymes involved in the degradation of the aldehyde via the corresponding fatty acid and β oxidation were expressed, indicating complete degradation of the alkyl group of BZK (see Fig. S3).

## DISCUSSION

BCC bacteria have the ability to remain viable for many months under harsh conditions, including organic solvents, antiseptics, and low nutrients (17). *Burkholderia* species have been reported to be capable of survival in water for prolonged periods of time (43–47). Previously, we reported that *B. cenocepacia* can persist in distilled water for >40 days (48). Also, BCC bacteria can remain viable with low susceptibility to antiseptics for a long time (49). In this investigation, some of the BCC strains persisted in the presence of 64 to 256 µg/ml BZK, C_14_BDMA-Cl, or C_12_BDMA-Cl, and 128 to 1,024 µg/ml C_10_BDMA-Cl. Fazlara and Ekhtelat (50) evaluated the susceptibility of six foodborne pathogens, including three Gram-positive and three Gram-negative bacteria, to BZK. *Listeria monocytogenes* and *Bacillus cereus* were the most sensitive and resistant bacteria studied, with MICs/minimum bactericidal concentrations (MBCs) equal to 30/35 µg/ml and 140/160 µg/ml, respectively. In our investigation, BCC strains were less susceptible to BZK (64 to 256 µg/ml) than clinical (methicillin-resistant) *Staphylococcus aureus* (51), *Listeria monocytogenes* (52), and *Salmonella enterica* serovar Typhimurium (53) isolates included in previous studies. Interestingly, *B. cenocepacia* showed levels of susceptibility to BZK (64 to 256 µg/ml), C_14_BDMA-Cl (64 to 256 µg/ml), and C_12_BDMA-Cl (64 to 256 µg/ml) that were twice as high as those of the other BCC strains. These results are in agreement with previous studies demonstrating a high MIC of BZK for clinical *B. cenocepacia* isolates (49, 54). *B. cenocepacia* was the most prevalent BCC species encountered in a recent survey of samples from cystic fibrosis and other clinical patients (54).

BZK acts on membrane permeability, causing leakage of cytoplasmic materials at low concentrations with damage to the outer membrane, and promotes its own intracellular uptake and entry (39, 55, 56). Because BZK enters BCC cells quickly by simple diffusion, the abilities of these strains to increase efflux pump activity may partly explain their resistance. The observations that CCCP, which inhibits transport, affected ethidium bromide accumulation (data not shown) and affected bacterial growth with C_12_BDMA-Cl indicates the involvement of the efflux pump in protecting *B. cenocepacia*. Ethidium bromide and MIC with CCCP were measured to evaluate efflux pump activity. The MIC of C_12_BDMA-Cl was 128 µg/ml without CCCP and 64 µg/ml with CCCP. These results provide indirect evidence that efflux pumps of BCC bacteria play a role in their survival in the presence of antiseptics (20, 23, 24).

Twenty BCC strains grew well in 1/10-diluted TSB medium supplemented with BZK, C_12_BDMA-Cl, or C_10_BDMA-Cl and degraded 2.9 to 66.1% of these compounds. One mechanism of resistance of BCC bacteria to BZK, C_14_BDMA-Cl, C_12_BDMA-Cl, and C_10_BDMA-Cl is degradation. Three QAC biodegradation pathways have been observed (31, 32, 36, 37, 57). So far, only one BZK biodegradation pathway, which begins with the fission of the central C-N bond and yields BDMA, has been reported (31, 36, 37, 41). In this study, BDMA and BMA were detected during the degradation of C_12_BDMA-Cl, indicating the initial oxidative cleavage of the central C-N bond (Fig. 2). On the other hand, C_6_BDMA-Cl, with low antiseptic activity toward BCC bacteria (survival at 1,024 µg/ml), was not degraded for about 40 days. This observation clearly indicates that C_6_BDMA-Cl is not a substrate for the enzyme catalyzing the initial oxidative C-N cleavage of C_12_BDMA-Cl. In addition, the low pleiotropic activity of the C-N cleavage enzyme(s) toward BZK could be associated with its (or their) substrate specificity governed by the alkyl chain length.

Proteomic data supported the observed efflux activity and biodegradation in terms of BZK resistance of BCC bacteria. In addition, the integrated view of the proteomic data with the genome further suggests the innate ability of *B. cenocepacia* to resist the activity of BZK; such natural insensitivity is due to (i) extrusion of BZK by chromosomally encoded active efflux pumps and (ii) innate production of metabolic enzymes that can degrade BZK. In this respect, the observed BZK resistance of all 20 of the BCC strains tested might be explained by the intrinsic resistance of BCC bacteria, which might be shared vertically. Considering their relatively high level of BZK resistance (>64 µg/ml), the intrinsic resistance mechanisms in BCC bacteria, efflux activity and biodegradation, seem to simultaneously work together to accelerate the development of high resistance levels.

The resistance of *B. cenocepacia* to toxic compounds is greatly dependent on the role of efflux pumps and works in synergy with reduced outer membrane permeability (20, 23–29). Especially, the direct functional evidence of the practical contribution to antibiotic resistance of efflux pumps of the RND and MFS families and the DsbA-DsbB disulfide bond formation system has been provided from the corresponding mutants generated by reverse genetic approaches (20, 26–29, 58). In this respect, control of membrane permeability and efflux pumping might be a primary response of BCC bacteria to BZK. Consistent with this common theme, two RND family transporters (Bcen_3015 and Bcen_4471), two MFS family transporters (Bcen_0548 and Bcen_4318), and the DsbB component of the DsbA (Bcen_2307)-BsbB (Bcen_0542) system were BZK-dependently regulated (STEM pattern 8), indicating their direct functional contribution to the BZK resistance of BCC bacteria. In addition, the transporter proteins belonging to other families, which showed apparent upregulation (STEM patterns 5 and 8), might also contribute to the BZK resistance of BCC bacteria in probably indirect ways. For example, the component Bcen_2284 of an ABC family transporter system with substrate specificity for aromatic compounds, such as toluene, showed STEM pattern 5 and no initial expression change (at day 3) but later (at day 7) upregulation. As discussed below, aromatic compounds possessing one benzene ring, including the toxic compound catechol, are metabolic intermediates of the biodegradation of BZK by BCC bacteria. On the other hand, when considering porin-mediated membrane permeability, the expression pattern of porins that act as molecular filters for hydrophilic compounds is intriguing. Indeed, the STEM-filtered 13 porins showed very diverse expression patterns (STEM patterns 1, 2, 3, 5, and 8), in which more than half of them were even upregulated. Therefore, this complex regulation of the porin proteins suggests that porin-mediated membrane permeability is not simple in the BZK resistance of BCC bacteria.

From toxicologic and metabolic perspectives, the cleavage (N dealkylation) of the C-N bond of BZK could be the most beneficial degradation route for *B. cenocepacia*. Apparently, N dealkylation of BZK dramatically decreases its toxicologic activity (see Fig. S2). In addition, despite the downregulation or nonexpression of many metabolic enzymes (C, E, F, G, H, I, P, and Q in COG), a set of constitutively expressed catabolic enzymes is able to completely further degrade the N dealkylation product BDMA and the aldehyde (or alcohol) to produce acetyl-CoA, a common intermediate of the central metabolism. Generation of acetyl-CoA from BZK by BCC bacteria has several metabolic significances, i.e., (i) complete degradation of BZK with no accumulation of the intermediate(s), including a toxic metabolite(s); (ii) metabolic connection between the peripheral BZK degradation pathway and the central carbon pathway; and (iii) utilization of BZK as a carbon and energy source. Therefore, N dealkylation-dependent degradation of BZK by *B. cenocepacia* is not only an effective detoxification process but also appears to be a beneficial metabolic behavior for using an alternative energy and carbon source.

Bacterial C-N bond cleavage of BZK is carried out by enzymes belonging to different families (40, 59). On the basis of the bibliomic, genomic, and proteomic data, among the candidate enzymes able to break the C-N bond of BZK, an amine oxidase (Bcen_5179) and a Rieske-type oxygenase (Bcen_4588) have been proposed for the initial cleavage. As reported in other studies (30, 31, 36, 37), interestingly, the BCC strains tested also showed apparent alkyl chain length-dependent C-N cleavage ability. Alkyl chain length dependence in terms of enzyme activity and substrate specificity is a typical specificity of *n*-alkane monooxygenase for initial terminal hydroxylation of *n*-alkanes (60). However, the gene (Bcen_0501) encoding an alkane monooxygenase has not been expressed. Further biochemical studies of the proposed C-N cleavage enzymes could be necessary in order to obtain direct evidence of the enzyme’s activity and substrate specificity.

The *n*-alkanes and aromatic compounds are chemically rather inert and should be activated before they are metabolized. In the perspective of the energy-costly activation steps, the biodegradation of the alkyl and aromatic metabolites generated by N dealkylation of BZK seems not to be problematic, although some of the enzymes responsible for the initial activation steps were missing. In the case of alkane degradation, since the N dealkylation of BZK generates an oxidized (aldehyde or alcohol) long alkyl chain, the initial terminal hydroxylation and the second oxidation of the primary fatty alcohol to the aldehyde are not necessary (60). In the case of BDMA, the aromatic compound could be completely degraded via benzoate, which should be oxygen-dependently activated by a Rieske-type ring-hydroxylating oxygenase(s) (61–63). Genome-scale screening has annotated three genes, Bcen_1304, Bcen_1305, and Bcen_1306, for a benzoate-hydroxylating oxygenase system. However, no expression of the ring-hydroxylating oxygenase system has been observed. As mentioned above, another Rieske-type oxygenase (Bcen_4588), a member of the ring-hydroxylating oxygenases with notorious pleiotropic activity toward diverse aromatic substrates, has been expressed (62–64).

Overall, the susceptibility of 20 BCC strains to BZK, C_14_BDMA-Cl, C_12_BDMA-Cl, C_10_BDMA-Cl, C_6_BDMA-Cl, and BTMA-Cl was determined. BCC bacteria showed low susceptibility (1,024 µg/ml) to C_6_BDMA-Cl and BTMA-Cl but high susceptibility (64 to 256 µg/ml) to C_14_BDMA-Cl, C_12_BDMA-Cl, and C_10_BDMA-Cl. *B. cenocepacia* HI2976 could not degrade all BZK compounds in distilled water. The presence of additional nutrients is necessary for degradation of BZK by *B. cenocepacia*. All of the BCC strains tested showed degradation ability and resistance to BZK. The two main resistance mechanisms, which are intrinsic and widespread, are extrusion of BZK by efflux pumps and biodegradation of BZK by constitutively expressed catabolic enzymes (Fig. 6). Information on the susceptibility, biodegradation, and intrinsic resistance mechanisms of BCC bacteria may be used to evaluate their ability to survive in BZK.

## MATERIALS AND METHODS

### Chemicals.

BZK, C_14_BDMA-Cl, C_12_BDMA-Cl, decyldimethylbenzylammonium chloride (C_10_BDMA-Cl), hexyldimethylbenzylammonium chloride (C_6_BDMA-Cl), benzyltrimethylammonium chloride (BTMA-Cl), BDMA, BMA, BA, benzaldehyde, and sodium perchlorate were obtained from Sigma-Aldrich Chemical Company, Inc. (St. Louis, MO). Acetonitrile was purchased from Fisher Scientific Company LLC (Pittsburgh, PA).

### Test organisms and growth conditions.

The 20 BCC strains utilized in this study are listed in Table 1. All were obtained from the *Burkholderia cepacia* Research Laboratory and Repository at the University of Michigan (48, 49). All BCC bacteria were routinely cultured on 1/10-diluted tryptic soy agar (TSA) and 1/10-diluted TSB at 23°C without shaking.

### Susceptibility of BCC strains to BZK and alkyl derivatives.

The MICs of BZK, C_14_BDMA-Cl, C_12_BDMA-Cl, C_10_BDMA-Cl, C_6_BDMA-Cl, and BTMA-Cl for BCC bacteria were tested in a 96-well microtiter plate and monitored in a Synergy MX spectrophotometer (BioTek Instruments, Winooski, VT) (49, 54). BZK and alkyl derivative dilutions of 8, 16, 32, 64, 128, 256, 512, and 1,024 µg/ml were prepared in 200 μl of Mueller-Hinton broth. The wells were inoculated with approximately 1.1 × 10^5^ CFU/ml, and the plates were incubated for 7 days at 23°C. Duplicate control wells, containing media without compounds, were inoculated with the same number of cells. Also, sterile medium spiked only with BZK served as abiotic degradation controls. After 7 days, turbidity (optical density at 600 nm [OD_600_]) was evaluated as the indicator of bacterial growth, and then the MIC of each chemical for BCC strains was determined as the minimum chemical concentration beyond which there was no bacterial growth. For determination of the MBC, samples in the MIC range were transferred to both 1/10-diluted TSA and 1/10-diluted TSB and evaluated for growth after 72 h (49).

### Effects of efflux pump inhibitors on BZK.

To evaluate the efflux pump activity of BCC bacteria, the kinetics of growth were monitored with the efflux pump inhibitor CCCP (Sigma) in a Synergy MX spectrophotometer (BioTek Instruments, Winooski, VT). Equal cell inoculations (final cell concentration of approximately 1 × 10^5^ CFU/ml) in 50 ml of 1/10-diluted TSB containing 60 µg/ml C_12_BDMA-Cl or 1 µg/ml CCCP were added to 250-ml Erlenmeyer flasks. The cultures were incubated at 23°C for 7 days, and cell growth was measured as OD.

### Degradation of BZK and alkyl derivatives.

Percentages of BZK, C_12_BDMA-Cl, and C_10_BDMA-Cl degradation by 21 BCC strains were tested in a 20-ml test tube. Dilutions of 32, 64, and 128 µg/ml BZK were prepared in 10 ml of 1/10-diluted TSB medium. The tubes were inoculated with approximately 1.1 × 10^5^ CFU/ml and incubated for 7 days at 23°C. After 7 days, 2-ml samples were extracted with 2 ml of acetonitrile and 2 ml of ethyl acetate (1:1, vol/vol) for 4 h. The acetonitrile-ethyl acetate extracts were pooled, dried, and reconstituted with 100 μl of acetonitrile prior to high-performance liquid chromatography (HPLC) analysis. The remaining concentrations of BZK were measured, and percent degradation was calculated. Experiments were done in triplicate, and mean values are shown.

The BZK and alkyl derivative analysis was performed by a modified method as described previously (41). Briefly, samples were analyzed by HPLC (Agilent 1200 series; Agilent Technologies, Inc., Wilmington, DE) with a C_18_ Luna SCX column (4.6 by 250 mm, 5 μm-particle size; Phenomenex, Torrance, CA) with UV detection at 265 nm. The initial mobile phase composition was 30% mobile phase A (20 mM sodium perchlorate in water) at a flow rate of 0.5 ml/min. Solvent B (20 mM sodium perchlorate in acetonitrile) was increased from 30 to 80% over 30 min and then held at 90% for 10 min.

The analysis of C_12_BDMA-Cl metabolites in *B. cenocepacia* AU1054 was performed with 5-ml cultures incubated in 1/10-diluted TSB without shaking for 7 days at 23°C. Liquid-liquid extraction was performed with 5 ml of sample, 2.5 ml of saturated NaHCO_3_, and 5 ml of methylene chloride. The organic phase was dried with sodium sulfate, taken to dryness, and reconstituted with 500 µl of acetonitrile prior to MS analysis. Separation was achieved with a Waters Acquity UPLC system (Waters Co., Milford, MA) with a 2.1- by 50-mm, 1.7-µm Acquity BEH HILIC column (Waters Co., Milford, MA) held at 40°C and eluted at 0.8 ml/min. Mobile phase A was 10 mM ammonium acetate, and mobile phase B was acetonitrile. Gradient conditions were as follows: 98% B to 50% B in 10 min, hold at 50% B for 5 min, and return to 98% B in 1 min. A Xevo TQ-S triple quadrupole mass spectrometer (Waters Co.) equipped with an electrospray interface operating in positive ion mode was used for all analyses. Positive ions were acquired in MRM mode. The transitions acquired for BDMA and BMA were as follows: 135.86 > 44.07 and 135.86 > 65.00 for BDMA and 122.02 > 65.00 and 122.02 > 91.07 for BMA.

### Proteome analysis.

We analyzed two biological replicates of a BCC whole-cell proteome at three time points (time zero, day 3, and day 7). The entire experimental procedures for proteome analysis were basically as described in our previous proteome studies (61, 64), except for the initial methods used for bacterial cell lysis and protein extraction. Briefly, cell pellets of BCC bacteria at each time point were resuspended in SDS-containing lysis buffer (100 mM Tris-HCl [pH 8.0], 2% SDS, 30% glycerol, 0.01% bromophenol blue) and then boiled for 10 min. Cell lysates were then quantified, and 20 μg of each lysate was subjected to 4 to 12% SDS-PAGE (Invitrogen, Carlsbad, CA). After electrophoresis, gels were excised into 40 equal bands per lane and, with a ProGest robot (DigiLab, Marlborough, MA), gel slices were further processed by gel washing, peptide reduction and alkylation, and peptide digestion. For MS analysis, gel supernatants were processed by nano-LC–tandem MS with a Waters NanoAcquity HPLC system interfaced with a Thermo (Fisher) Q Exactive mass spectrometer. We used a local copy of Mascot for data searching with the parameters described previously (61). Mascot DAT files were parsed into the Scaffold software for validation, filtering, and creation of a nonredundant list per sample. Data were filtered at a 1% protein and peptide false discovery rate and required at least two unique peptides per protein. Spectral counts were exported to Excel, and data were normalized by using normalized spectral abundance factor (NSAF) values. A *t* test was performed in Excel, and fold change was calculated on the basis of the mean NSAF value of each group. Expression of proteins was determined as significantly different on the basis of two criteria, (i) a protein *P* value of <0.05 and (ii) protein detection with a ≥2-fold (up or down) change, based on division of the NSAF values. For detailed information on proteome analysis and statistics, see reference 61.

## SUPPLEMENTAL MATERIAL

Figure S1  Kinetics of growth (OD_600_) of *B. cenocepacia* HI2976 measured in medium with different concentrations of BZK (A), C_14_BDMA-Cl (B), C_12_BDMA-Cl (C), C_10_BDMA-Cl (D), C_6_BDMA-Cl (E), and BTMA-Cl (F). Symbols represent averages of triplicate values from three samples, and error bars represent the standard deviations. Download Figure S1, PDF file, 0.1 MB

Figure S2  Possible pathways of BZK degradation by *B. cenocepacia* strain AU1054. Download Figure S2, PDF file, 0.1 MB

Figure S3  Possible pathway of benzoate degradation by strain AU1054. Download Figure S3, PDF file, 0.1 MB

Table S1 Proteomic data of strain AU1054. Table S1, XLSX file, 2.5 MB

Table S2 STEM analysis of strain AU1054 proteomic data. Table S2, XLSX file, 0.2 MB

Table S3 STEM analysis of transporters of strain AU1054. Table S3, XLSX file, 0.1 MB
