# Supplementary material for: Intrinsic Resistance of Burkholderia cepacia Complex to Benzalkonium Chloride
Source: mBio. 2016 Nov 22;7(6):e01716-16. doi: 10.1128/mBio.01716-16 (PMC5120141; doi:10.1128/mBio.01716-16)
Supplement: Figure S1 — Kinetics of growth (OD600) of B. cenocepacia HI2976 measured in medium with different concentrations of BZK (A), C14BDMA-Cl (B), C12BDMA-Cl (C), C10BDMA-Cl (D), C6BDMA-Cl (E), and BTMA-Cl (F). Symbols represent averages of triplicate values from three samples, and error bars represent the standard deviations. Download [file mbo006163084sf1.pdf]

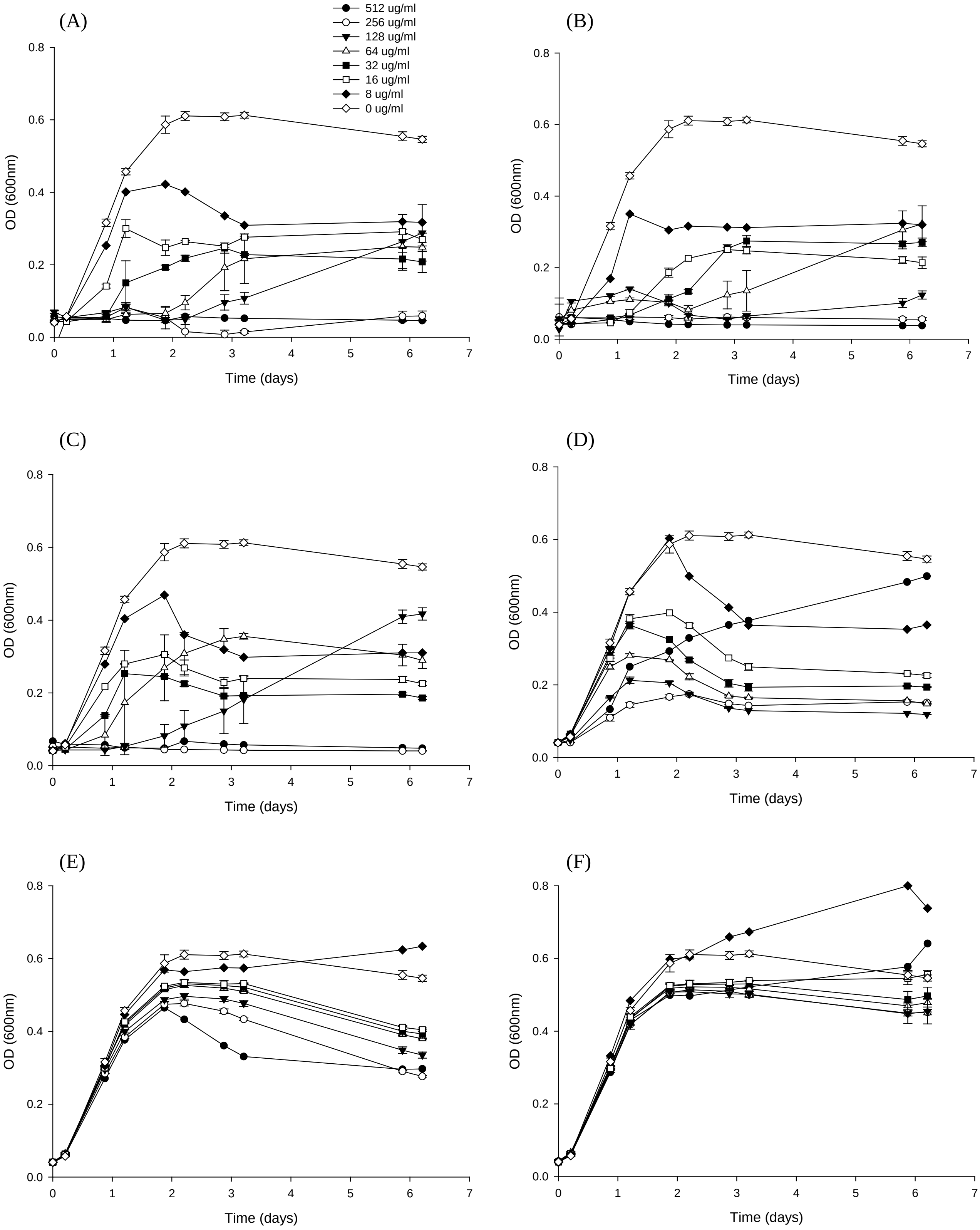

Fig. S1. Kinetics of growth (OD_{600}) of *B. cenocepacia* HI2976 were measured in the BZK(A), C_{14}BDMA-Cl(B), C_{12}BDMA-Cl(C), C_{10}BDMA-Cl(D), C_6BDMA-Cl(E), and BTMA-Cl(F) in media with different concentrations. Symbols represent averages of triplicates from three samples and error bars represent the standard deviations.
