# Supplementary figures and images for: Intrinsic Resistance of Burkholderia cepacia Complex to Benzalkonium Chloride
Source: mBio. 2016 Nov 22;7(6):e01716-16. doi: 10.1128/mBio.01716-16 (PMC5120141; doi:10.1128/mBio.01716-16)

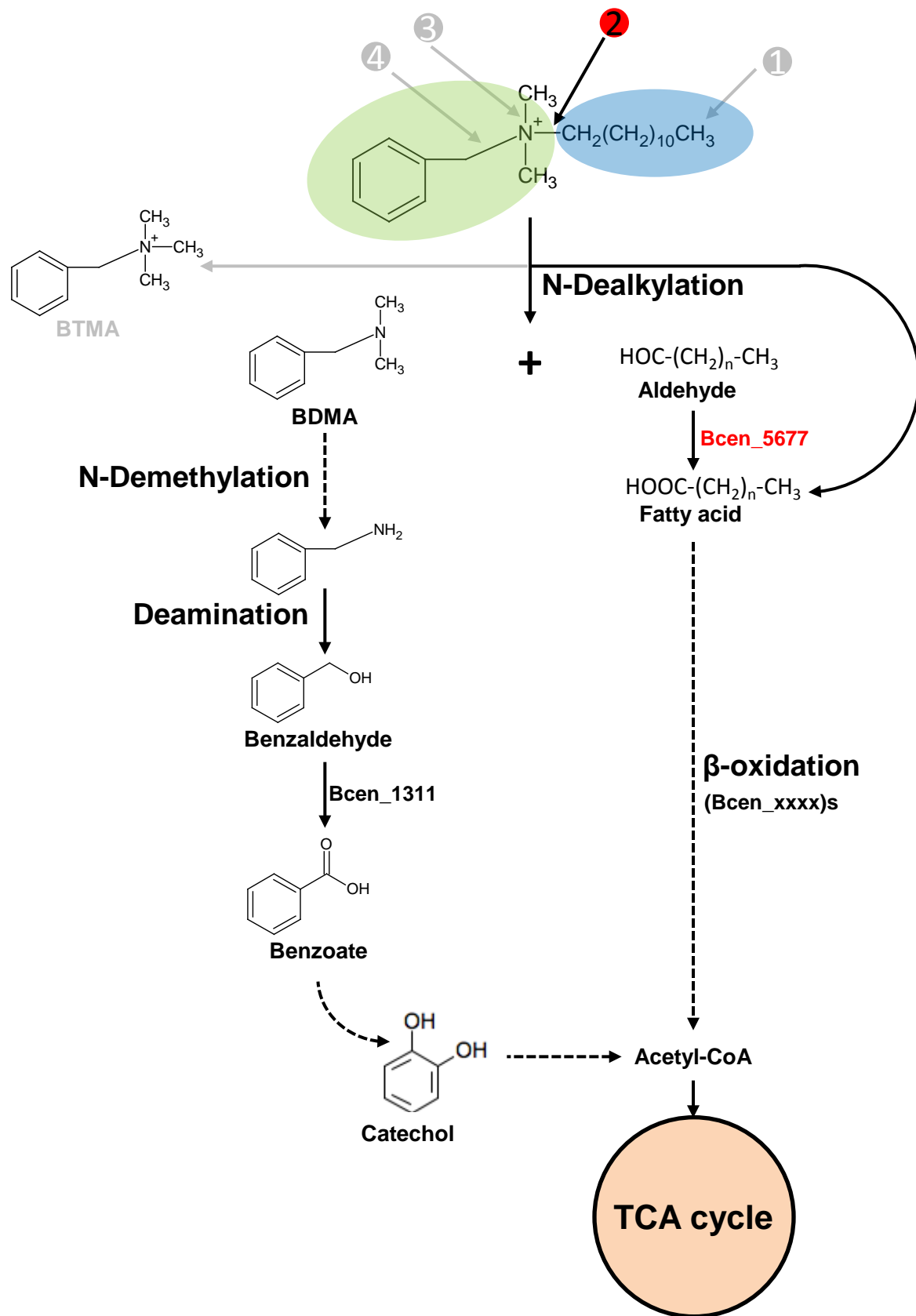

Fig. S2. Possible degradation pathways of BZK in strain AU1054

Supplement: Figure S2 — Possible pathways of BZK degradation by B. cenocepacia strain AU1054. Download [file mbo006163084sf2.pdf]

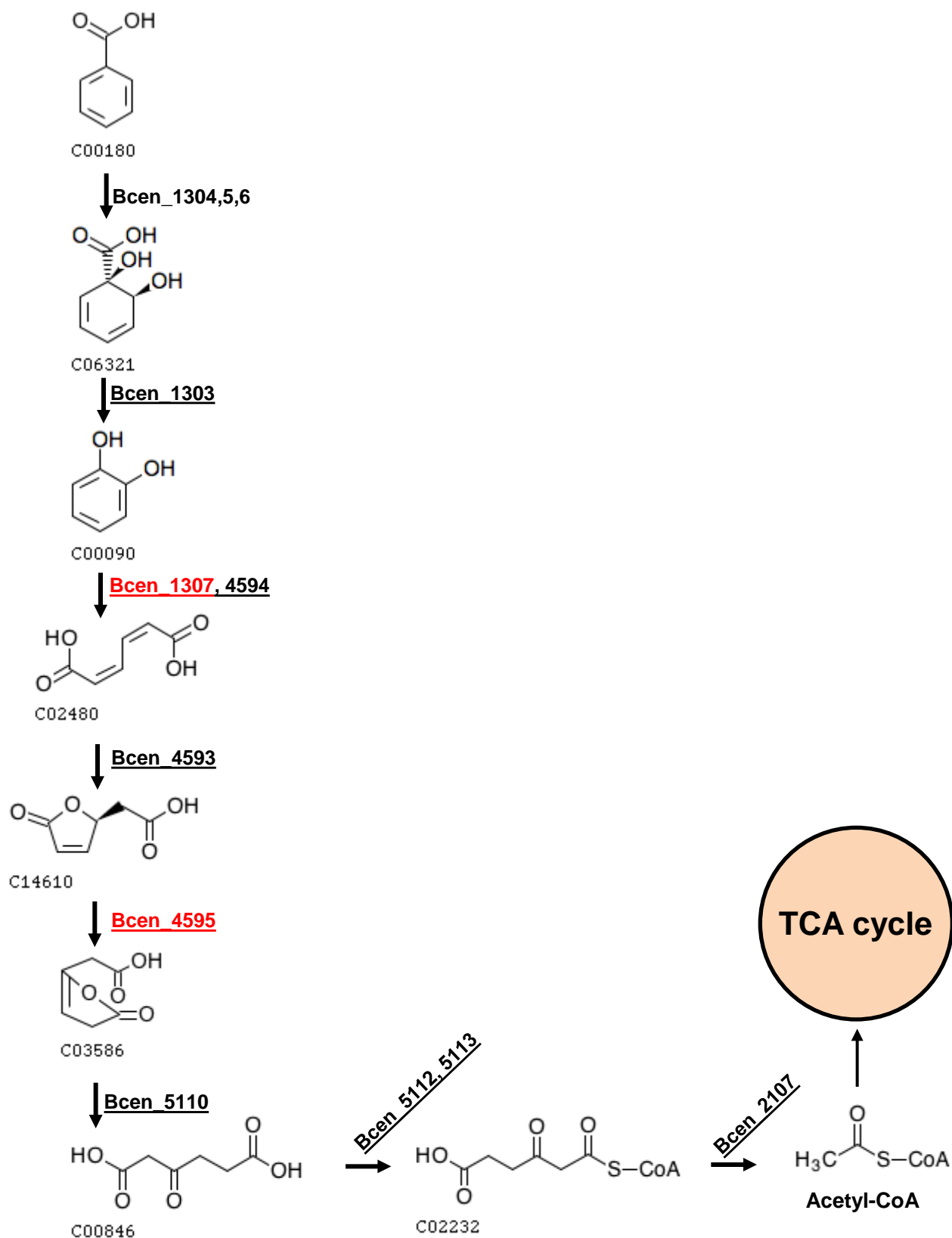

Fig. S3. Possible degradation pathway of benzoate in strain AU1054

Supplement: Figure S3 — Possible pathway of benzoate degradation by strain AU1054. Download [file mbo006163084sf3.pdf]
